# Ganglioside GD2-specific trifunctional surrogate antibody Surek demonstrates therapeutic activity in a mouse melanoma model

**DOI:** 10.1186/1479-5876-10-219

**Published:** 2012-11-07

**Authors:** Peter Ruf, Beatrix Schäfer, Nina Eissler, Ralph Mocikat, Juergen Hess, Matthias Plöscher, Susanne Wosch, Ivonne Suckstorff, Christine Zehetmeier, Horst Lindhofer

**Affiliations:** 1 TRION Research GmbH, Martinsried, Germany; 2 Helmholtz-Zentrum München, Institut für Molekulare Immunologie, Munich, Germany; 3 TRION Pharma GmbH, Munich, Germany; 4 Department of Antibody Development, TRION Research GmbH, Am Klopferspitz 19, 82152, Martinsried, Germany

**Keywords:** Immunotherapy, Trifunctional bispecific asntibody, Ganglioside GD2, Melanoma

## Abstract

**Background:**

Trifunctional bispecific antibodies (trAb) are a special class of bispecific molecules recruiting and activating T cells and accessory immune cells simultaneously at the targeted tumor. The new trAb Ektomab that targets the melanoma-associated ganglioside antigen GD2 and the signaling molecule human CD3 (hCD3) on T cells demonstrated potent T-cell activation and tumor cell destruction *in vitro*. However, the relatively low affinity for the GD2 antigen raised the question of its therapeutic capability. To further evaluate its efficacy *in vivo* it was necessary to establish a mouse model.

**Methods:**

We generated the surrogate trAb Surek, which possesses the identical anti-GD2 binding arm as Ektomab, but targets mouse CD3 (mCD3) instead of hCD3, and evaluated its chemical and functional quality as a therapeutic antibody homologue. The therapeutic and immunizing potential of Surek was investigated using B78-D14, a B16 melanoma transfected with GD2 and GD3 synthases and showing strong GD2 surface expression. The induction of tumor-associated and autoreactive antibodies was evaluated.

**Results:**

Despite its low affinity of approximately 10^7^ M^-1^ for GD2, Surek exerted efficient tumor cell destruction *in vitro* at an EC_50_ of 70ng/ml [0.47nM]. Furthermore, Surek showed strong therapeutic efficacy in a dose-dependent manner and is superior to the parental GD2 mono-specific antibody, while the use of a control trAb with irrelevant target specificity had no effect. The therapeutic activity of Surek was strictly dependent on CD4^+^ and CD8^+^ T cells, and cured mice developed a long-term memory response against a second challenge even with GD2-negative B16 melanoma cells. Moreover, tumor protection was associated with humoral immune responses dominated by IgG2a and IgG3 tumor-reactive antibodies indicating a Th1-biased immune response. Autoreactive antibodies against the GD2 target antigen were not induced.

**Conclusion:**

Our data suggest that Surek revealed strong tumor elimination and anti-tumor immunization capabilities. The results warrant further clinical development of the human therapeutic equivalent antibody Ektomab.

## Background

The tumor-associated ganglioside GD2 is an attractive target for immunotherapy. While its expression in normal tissue is restricted to the central nervous system and peripheral nerves [1,2] it is strongly detectable on neuroblastoma (NB) and on most melanoma lesions [3,4]. Additionally, it is found on sarcoma, glioma and in approximately 50%-100% of small cell lung cancers (SCLC) where it is associated with enhanced cell proliferation and invasive activity [5-7]. Due to its distribution pattern, GD2 has been chosen as a target for monoclonal antibody therapy. Early clinical trials indicated certain efficacy especially in the treatment of NB [8]. Recently, the GD2-specific chimeric antibody ch14.18 in combination with IL-2 and GM-CSF demonstrated improved overall survival in high risk NB patients as compared to standard therapy (isotretionin) alone [9]. Undoubtedly, this study validates GD2-targeted immunotherapy for NB patients without bulky disease. However, treatment of other solid GD2-positive tumors like melanoma has shown limited clinical success so far [10,11]. A promising new approach might be provided by genetically engineered T cells expressing GD2-specific chimeric antigen receptors, which demonstrated anti-melanoma activity in a xenograft model [12].

Alternatively, GD2-targeting bispecific molecules may be applied to recruit T cells to the tumor thereby avoiding T-cell manipulation *ex vivo*. Trifunctional bispecific antibodies (trAb) represent a new class of T cell-recruiting immunotherapeutics with enhanced effector functions. They consist of a binding arm directed against a tumor-associated antigen (TAA), a second binding arm specific for CD3 on T cells and a chimeric mouse IgG2a x rat IgG2b Fc region that shows preferential binding of activating Fcγ receptors (FcγR) expressed on monocytes, macrophages, dendritic and natural killer cells [13,14]. TrAb induce a concerted and highly efficient attack against tumor cells by redirecting different types of immune effector cells as shown *in vitro*[15-17] and *in vivo*[18-20]. In 2009, with catumaxomab (anti-EpCAM x anti-CD3), the first bispecific antibody worldwide was approved in the European Union for the treatment of malignant ascites in patients with EpCAM-positive tumors [21]. This motivated the development of further trAb designed for other cancer entities such as melanoma. A GD2-specific trAb (TRBs07/Ektomab) demonstrated significant cytotoxic potential against melanoma cells *in vitro*[22]. However, the relatively low GD2 affinity of this trAb was a matter of concern and raised the question of its *in vivo* efficacy. Therefore, we developed the surrogate trAb Surek that consists of the identical anti-GD2 binding arm but targets mouse instead of human CD3. Thus, Surek can be used in experimental tumor models using immune competent mice for the treatment of GD2-positive malignant disease. Here, we report on the characterization of this surrogate antibody and on its effective *in vivo* application as a preclinical research biologic.

## Methods

### Manufacture and quality control of Surek and control antibodies

The trAb Surek (anti-GD2 x anti-mouse CD3), its parental control antibodies Me361 [23] (anti-GD2; mouse IgG2a/kappa), 17A2 [24] (anti-mouse CD3; rat IgG2b/kappa), and its therapeutic homologue Ektomab/TRBs07 [22] (anti-GD2 x anti-human CD3) and the control trAb TRBs01 (anti-HER2/neu x anti-mouse CD3) were produced by quadroma or hybridoma cells according to the TRION antibody platform technology as described [13]. For the manufacture, chemically defined protein-free medium was used (Invitrogen, USA). Endotoxin content of purified antibody stock solutions was measured by Limulus amebocyte lysate (LAL) gel clotting test (Pyroquant Diagnostik, Germany). Monomer and aggregate distribution was determined by size exclusion (SE) – HPLC (HP 1100 system, Agilent, USA) using a TSKgel G3000SWXL column (Tosoh Biosep, USA). For reduced mass analysis and determination of the peak area ratio of the antibody chains, Surek samples were denatured by using 6M guanidine, reduced with dithiothreitol and alkylated with iodacetamid. The samples were analyzed by means of RP-HPLC ESI-TOF-MS (Agilent 1200 online coupled with an Agilent 6220 ESI-TOF, CA, USA) using a 250 mm x 2 mm Jupiter C5 column, packed with 5 μm particles, 300 Å pore size (Phenomenex, Torrance, CA, USA). The raw mass spectra of the antibody chains were converted using MassHunter software to calculate the observed masses. The reversed phase chromatogram with UV absorbance at 214 nm was used for the determination of the peak area ratio. Bispecific binding activity of Surek and Ektomab was evaluated by flow cytometry with a FACSCalibur (Becton Dickinson, USA). GD2-positive B78-D14 [25] and mouse CD3-positive LBRM-33 (ATCC: TIB-155) cells served as targets. Cell-bound trifunctional bispecific antibodies were either detected by FITC-labelled anti-mouse IgG or anti-rat IgG secondary antibodies (Dianova, Germany). The GD2-specific control antibody 14G2a [26] was purchased from Santa Cruz Biotechnology (CA, USA).

### Antibody binding to GD2 and GD3

Relative binding affinity of Surek to the gangliosides GD2 and GD3 was measured by ELISA. Briefly, ELISA plates (high binding, Greiner bio-one, Germany) were coated with 0.2 μg/well GD2 or GD3 (purified from human brain, Biomol, Germany) in ethanol, dried and blocked over night with SuperBlock blocking buffer (Pierce, USA). After washing with TRIS buffer at pH8, Surek and control antibodies Me361, 14G2a and Ektomab were added in PBS containing 4% bovine serum albumin at the indicated concentrations. After one hour, plates were washed and bound antibodies were detected with a mixture of biotin-conjugated F(ab^′^)_2_ anti-mouse/rat IgG specific detection antibodies (Jackson Immuno Research, USA). Then, streptavidin β-galactosidase and finally its corresponding substrate, chlorphenolred-β-D-galactopyranosid (Roche Diagnostics, Germany), were added, and the colorimetric reaction was measured at 570 nm by a VersaMax microplate reader (Molecular Devices, USA). Sigmoidal binding curves were five parameter fitted using GraphPad Prism software (version 5.02) and EC_50_ values were compared.

### Cytotoxicity assay

Antibody-mediated cellular cytotoxicity was measured by a colorimetric 2,3-bis[2-methoxy-4-nitro-5-sulfophenyl]-2H-tetrazolium-5-carboxanilide inner salt (XTT)-based assay. Briefly, GD2-positive B78-D14 target cells were coincubated with mouse effector cells in the presence of indicated antibody concentrations in 96-well flat-bottom plates. The effector to target ratio was 50:1. As effector cells we used spleen and lymph node cells of naïve C57BL/6 mice after enriching T cells in one round of B-lymphocyte panning with anti-IgG+M antibodies (Dianova, Germany). Moreover, macrophages derived from peritoneal lavage were added to increase the number of accessory immune cells. The effector cell population consisted of about 40% CD4^+^/CD3^+^ T-cells, 25% CD8^+^/CD3^+^ T cells, 10% CD11b^+^ macrophages/monocytes, 5% CD3^-^/NK1.1^+^ NK cells, and 15% remaining CD3^-^/CD19^+^ B-cells as determined by flow cytometry (data not shown). After three days, effector cells were removed by washing. Adherent viable tumor cells were stained with XTT cell proliferation kit II (Roche Diagnostics, Germany). Absorbance was measured in a Versamax microplate reader and % cell killing was calculated as follows: [(absorbance target cells - absorbance sample)/(absorbance target cells – absorbance effector cells)] x 100. Each sample was performed in quadruplicates. Surek Kiling curves of mean values were fitted using five parameter equation of GraphPad Prism software (version 5.02).

### Cells, mice and tumor models

The GD2-positive B78-D14 mouse melanoma cell line was derived from GD2-negative B16 cells by transfection with genes coding for the GD3 and GD2 synthases as described [25]. The cell line was a kind gift of Prof. J. C. Becker (Graz, Austria). Cells were cultivated in RPMI 1640 medium supplemented with 8.9% fetal calf serum, 2 mM L-glutamine, 1 mM sodium pyruvate, and 1 x nonessential amino acids. Additionally, 400 μg G418 and 500 μg Hygromycin B per ml were added to the B78-D14 cell culture. Cells were extensively washed in PBS before *in vivo* application.

C57BL/6J mice (Taconic, Denmark), 9 to 12 weeks of age, were injected i.p. with 1–5 × 10^5^ tumor cells followed by Surek or Me361 control antibody treatment as outlined. In depletion experiments, either CD8^+^ or CD4^+^ T cells were eliminated by injecting 0.5 mg of the cell-depleting antibodies RmCD4 or RmCD8 on day −4 and subsequently 0.1 mg on days 1 and 14. Long-term survivors of a primary tumor challenge were re-challenged 23 weeks later with 3 × 10^3^ GD2-negative B16 wildtype cells without any further treatment to assess anti-tumor immunity. In all experiments tumor control groups receiving tumors cells only were performed. For adoptive plasma transfer experiments 200 μl of immune or naïve control plasma were *ex vivo* mixed together with 3 × 10^3^ B16 tumor cells and i.p. administered into naïve mice. Mice were killed after apparent i.p. tumor growth (abdominal swelling) that was confirmed by dissection *post mortem*. For the assessment of tumor-reactive antibodies, blood samples were collected three weeks after Surek therapy of the primary tumor challenge and two weeks after tumor re-challenge. All animal experiments were in accordance with relevant regulations and had been approved by the local authority.

### Detection of tumor-reactive antibodies

Antibodies against whole tumor cells were measured by flow cytometry using either B78-D14 or B16-F0 as target cells. Mouse plasma samples were incubated at a final dilution of 1:30 with 2–4 × 10^5^ tumor cells for 60 min at 2-8°C. After two washing rounds, cell-bound antibodies were detected by a FITC-conjugated rat-anti-mouse IgG (Dianova, Germany) or by subclass-specific goat anti-mouse IgG1, IgG2a and IgG3 secondary detection antibodies (Southern Biotechnology, USA). Mean fluorescence intensity (MFI) and % of positively stained cells were determined with a FACSCalibur cytometer and corresponding CellQuest analysis program (Becton Dickinson, USA). Nonspecific (background) binding of secondary antibodies alone was below 5%. GD2-specific antibodies were quantified by ELISA. Therefore, mouse plasma samples were two-fold serially diluted on GD2-coated >ELISA plates and bound antibodies were detected by a mixture of biotin-conjugated rat anti-mouse IgG1, 2a, 2b and 3 specific antibodies (gift of E. Kremmer, Helmholtz-Zentrum München, Germany). The GD2-binding antibody Me361 was used as a positive control. Antibody titers were defined by the highest sample dilution with significant signal above buffer background (mean _background_ + 3 x SD).

### Statistical analysis

Differences in anti-antibody titers were statistically analyzed by unpaired, two-tailed Students *t*-test. Survival curves of mice were compared by log-rank test. *P* values < 0.05 were considered statistically significant. GraphPad Prism software version 5.02 (GraphPad Software) was used for generation of survival curves, titer plots and statistical calculations.

## Results

### Chemical and functional quality of the therapeutic antibody homologue Surek

**Figure 1:**
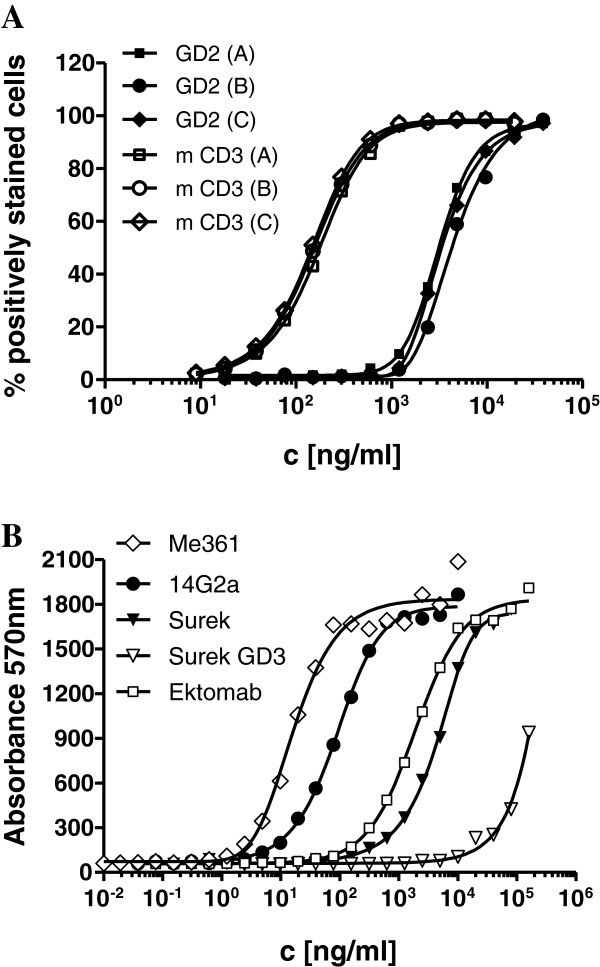
**Binding of Surek and control antibodies.** (**A**) Three different production batches A, B and C of Surek were compared for bispecific binding to either GD2 positive B78-D14 or to mouse CD3 expressing LBRM-33 cell lines by flow cytometry. In mean, binding to mouse CD3 (EC_50_ = 0.16 μg/ml [1.07 nM]) was 23 times stronger than binding to GD2 (EC_50_ = 3.6 μg/ml [24 nM]). (**B**) Binding curves of the monoclonal antibodies Me361, 14G2a, Surek and Ektomab to GD2 as measured by ELISA are displayed. Additionally, cross-reaction of Surek with GD3 is included. The figure shows one representative of three independent experiments. Mean calculated EC_50_ values were 0.018 μg/ml [0.12 nM] (Me361), 0.073 μg/ml [0.49 nM] (14G2a), 4.8 μg/ml [32 nM] (Surek) and 3.1 μg/ml [20.7 nM] (Ektomab).

The trifunctional surrogate antibody Surek was manufactured according to the antibody platform technology developed by TRION as described elsewhere [13]. Analysis of three production batches showed very low aggregate content (< 1%) and acceptable low endotoxin contamination of less than 10^2^ EU/ml. Reversed phase HPLC and mass spectrometric analysis confirmed the presence of all four mouse and rat Ig heavy and light chains in an almost ideally 1:1:1:1 proportion (not shown). Moreover, comparable bispecific binding to GD2 and mouse CD3 positive cell lines could be demonstrated for all three batches by flow cytometry (Figure 1A).

Next, we investigated the specificity and affinity of Surek for GD2 by ELISA. There was only weak cross reactivity with the most related ganglioside structure GD3 at therapeutically irrelevant high concentrations of >100 μg/ml (Figure 1B). Since both antibodies contain the identical anti-GD2 binding arm, Surek and its therapeutic equivalent Ektomab showed similar binding curves, as expected. Moreover, relative binding affinity for both monovalent antibodies was 40–70 times weaker as compared to the bivalent GD2-specific mouse antibody 14G2a whose association constant for cell-bound GD2 was determined with 1,5 × 10^8^ M^-1^[26]. Thus, Surek and Ektomab represent antibodies for the GD2 antigen with an estimated association constant in the 10^7^ M^-1^ range. Best binding results were obtained with parental antibody Me361 which bound approximately 4 times stronger in comparison to 14G2a.

**Figure 2:**
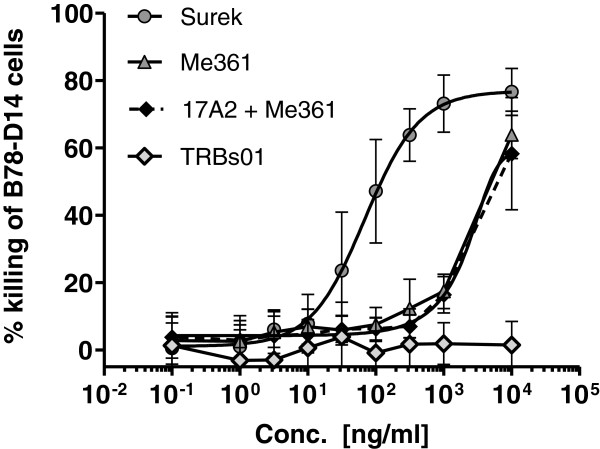
**Surek-mediated killing of GD2-positive B78-D14 melanoma cells by mouse immune effector cells.** Tumor cell killing was measured by an XTT-based long-term cytotoxicity assay using enriched T cells and peritoneal macrophages at an effector to target ratio of 50:1. Surek antibody mediated efficient killing of B78-D14 cells with a mean EC_50_ of 70 ng/ml [0.47 nM]. Anti-GD2 parental antibody Me361 was only effective at the highest tested concentration of 10,000 ng/ml; the addition of the second parental antibody 17A2 (anti-mouse CD3) did not improve killing activity, nor had the control trAb TRBs01 (anti-HER2/neu x anti-mCD3) any effect. The figure summarizes three independent experiments. Error bars indicate SD.

Finally, we characterized the functional activity of Surek by measuring the cytotoxicity against GD2-positive B78-D14 mouse melanoma cells in the presence of mouse effector T and accessory immune cells. In Figure 2, three independent experiments are summarized showing that Surek mediated effective elimination of tumor cells with a calculated mean EC_50_ of 70 ng/ml [0.47 nM]. In contrast, the parental anti-GD2 mouse antibody Me361 was only effective at the highest used concentration of 10,000 ng/ml, probably acting through the mechanism of ADCC as indicated previously [27]. The addition of the parental anti-CD3 antibody 17A2 did not increase Me361 cytotoxic activity indicating that nonspecific T-cell activation did not contribute to tumor cell destruction. This was further confirmed with the control trAb TRBs01 that cannot bind to the B78-D14 target cells and accordingly revealed no cytotoxicity. Of note, tumor cell killing activity of bsF(ab′)^2^ fragments was strongly impaired emphasizing the importance of the Fc part of Surek for its therapeutic efficacy (manuscript submitted).

### Dose- and T cell-dependent therapeutic effect of Surek *in vivo*

**Figure 3:**
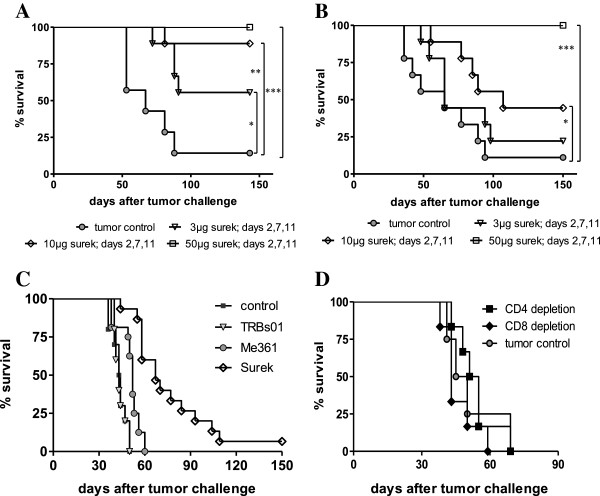
**Therapeutic effectiveness of Surek.** Survival of mice i.p. injected with different numbers of B78-D14 tumor cells followed by Surek therapy was evaluated. Mice were administered with either 1 × 10^5^ (**A**) or with 2 × 10^5^ (**B**) B78-D14 cells and subsequently received three injections of Surek antibody within 11 days, except for the tumor control. Three dosing groups of 3, 10 and 50 μg antibody were performed. Each group comprised 9 mice. Asterisks indicate statistical significance in comparison to the control group according to the log rank test with *p* values of < 0.05 (*), < 0.01 (**), and < 0.001 (***), respectively. (**C**) Superior therapeutic effect of Surek in comparison with the parental anti-GD2 antibody Me361 and the control trAb TRBs01 (anti-HER2/neu x anti-mCD3) was observed. Mice were challenged i.p. with 5 × 10^5^ B78-D14 cells and treated with three injections of either 50 μg Surek (n=15), 50 μg Me361 (n=16), or 50 μg TRBs01 (n=10) antibody, or left untreated (control, n=10). Survival of mice treated with Surek was significantly prolonged in comparison to the Me361 and TRBs01 treatment groups (log-rank test, *p* < 0.0001). (**D**) The therapeutic activity of Surek depends on the presence of T cells. Mice (n=6) were challenged i.p. with 1 × 10^5^ B78-D14 cells on day 0 and treated with 10 μg Surek on days 2 and 10 after tumor challenge. T cells were abrogated by injection of depletion antibodies RmCD4 and RmCD8 on days −4, 1 and 14.

We tested the *in vivo* efficacy of Surek by monitoring the inhibition of intraperitoneally (i.p.) growing B78-D14 tumor cells. Mice were inoculated either with 1 × 10^5^ or 2 × 10^5^ tumor cells and received three consecutive injections (i.p.) of Surek on days 2, 7 and 11 after tumor challenge. Three doses of 50 μg (high), 10 μg (middle) or 3 μg (low) per injection were given. Control animals received tumor cells but no antibody. The corresponding survival curves clearly demonstrate a dose-dependent therapeutic effect of Surek. In both experiments, tumor growth was completely impeded in all animals (100%) at the highest dose of 50 μg (Figure 3A, B). Survival rates of the low and middle dosing groups depended on the number of initially applied tumor cells. Prolongation of survival in comparison to the control groups, which showed 14% and 11% survivors, respectively, was significantly improved for all Surek treatment groups except for the lowest dosing group of 3 μg in combination with the high tumor cell burden.

Importantly, Surek significantly improved (*p* < 0.0001) the therapeutic outcome in comparison to the parental anti-GD2 monospecific antibody Me361. After application of 5 × 10^5^ tumor cells, the median survival time of mice was increased from 43.5 days (control) to 52 days by Me361 and further to 67 days by Surek (Figure 3C). This suggests that the therapeutic activity of Surek mainly results from T cells and that mono-specific antibody-mediated killing mechanisms like ADCC and CDC as observed for anti-GD2 antibody 14G2a [28] play a minor role. This assumption was confirmed by T-cell depletion experiments. *In vivo* depletion of CD8^+^ and of CD4^+^ T cells, respectively, completely abrogated the therapeutic effect of Surek indicating that both T cell subpopulations are required (Figure 3D). Finally, Surek-mediated tumor elimination was specific since the use of the control trAb TRBs01 with unrelated target specificity had no impact on the survival of the mice (Figure 3C).

### Long-term anti-tumor immunization induced after Surek therapy

**Figure 4:**
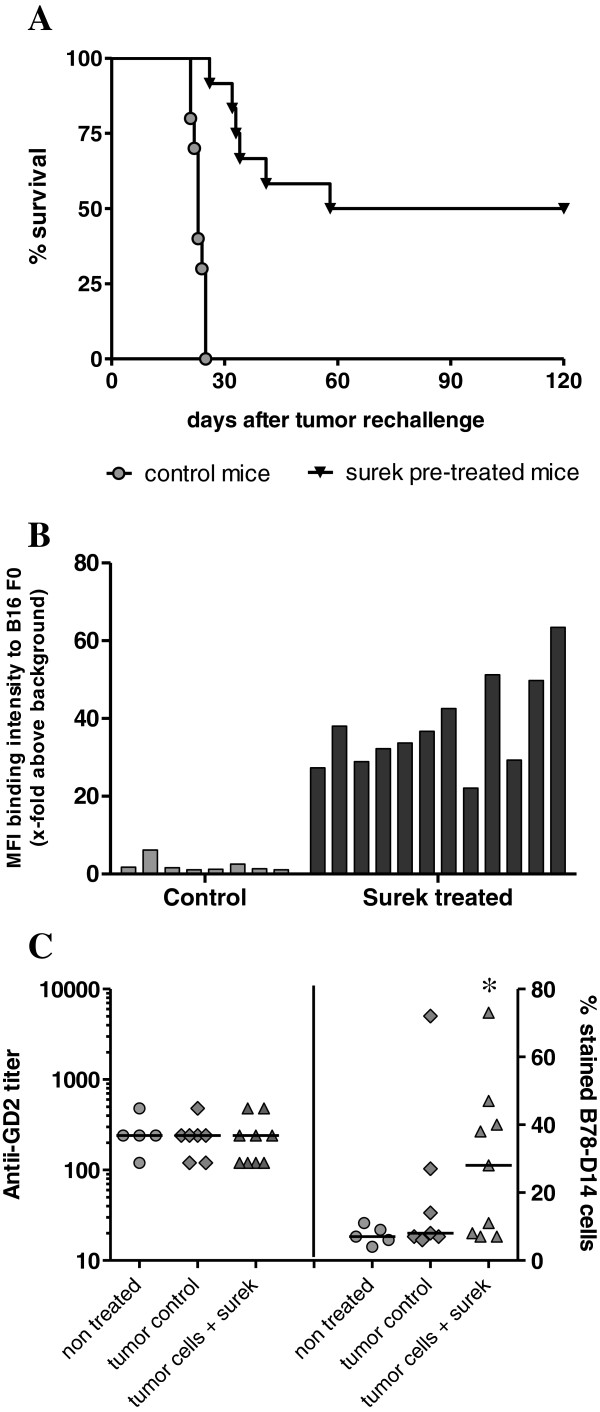
**Survival of mice after tumor rechallenge and induced humoral immune response.** (**A**) Long-term survivors were rechallenged (i.p.) with 3 × 10^3^ B16-F0 cells 23 weeks after Surek therapy. Six of 12 mice (50%) completely rejected the second tumor challenge in contrast to non pre-treated control mice (n = 10; log-rank test, *p* < 0.0001). Data of two independent experiments were summarized. (**B**) The binding of individual mouse plasma samples to B16 F0 cells was analyzed by FACS. Samples of long-term survivors (Surek pre-treated) or non-pretreated mice (control) were collected 14 days after tumor rechallenge with B16-F0 cells. The two analyzed sample groups are significantly different (*p* < 0.0001). (**C**) Induced tumor reactive antibodies are not directed against the target antigen GD2. Mice were i.p. treated as indicated in Figure 3 with 1 × 10^5^ B78-D14 tumor cells followed by 3 × 50 μg of Surek. Control animals were either left untreated or received only tumor cells. Three weeks after therapy, plasma samples were collected and analyzed for anti-GD2 (left panel) and for anti-B78-D14 antibodies (right panel). Only background anti-GD2 titers were detectable whereas a significant reaction with B78-D14 cells was observed in the group treated with tumor cells and Surek antibody as indicated by the asterisk (*p* = 0.024).

**Figure 5:**
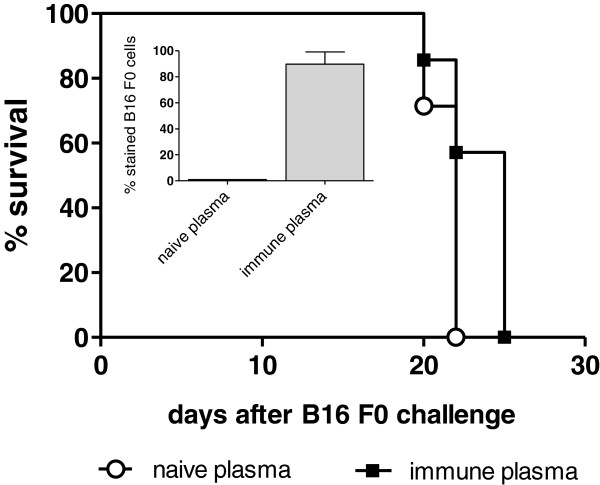
**Adoptive transfer of immune plasma.** The adoptive transfer of 200 μl of immune plasma simultaneously administered i.p. together with 3 × 10^3^ B16 F0 cells had only a marginal if any effect on tumor growth in comparison to the transfer of naïve plasma: Median survival of mice (n = 7) was 25 versus 22 days (log-rank test, *p* = 0.044). The inlet figure demonstrates that 90% of B16 F0 cells were stained positive by the mouse immune plasma. The experiment was once repeated with similar results.

Having shown that, in spite of its relatively low affinity, Surek efficiently eradicates GD2-positive melanoma cells *in vivo*, we addressed the question whether this antibody is also capable of inducing long-lasting anti-tumor immunization effects. Recently, tumor-protective immunization has been observed with the high affinity EpCAM-specific surrogate antibody BiLu [18,29]. Therefore, we re-challenged long-term survivors 23 weeks after Surek therapy with a lethal dose of B16F0 wild type melanoma cells. In contrast to non-immunized control animals, all of which developed tumors within 4 weeks, 50% of the immunized mice displayed tumor protection and completely rejected a tumor challenge (Figure 4 A, p< 0.0001). When we analyzed plasma samples taken 14 days after tumor re-challenge for tumor-reactive antibodies, we found a strong anti-B16F0 immune response in the Surek pre-treated group (Figure 4B). IgG subclass analyses indicated a Th1 response with dominant IgG3 and IgG2a but low IgG1 titers (Table 1). Interestingly, antibody responses were not directed against the target antigen GD2 as evaluated by ELISA: Plasma samples collected 3 weeks after Surek treatment were negative for GD2-specific antibodies (Figure 4C). On the other hand, 5 of 9 mice treated with Surek showed a clear response against whole B78-D14 tumor cells that was significant in comparison to the control group (*p* = 0.024). This demonstrates that Surek therapy induced tumor-reactive antibodies but did not evoke autoimmune reactions against the endogenous tumor-associated glycolipid GD2. Finally, by performing adoptive plasma transfer experiments we demonstrated that the tumor-reactive antibodies themselves had only marginal if any inhibitory effects on tumor growth. Median survival of immune plasma versus naïve plasma treated mice was 25 versus 22 days (Figure 5).

**Table 1:** IgG subclass analysis of tumor-reactive antibodies from long-term survivors

**Sample**	**Classification**		
	**IgG1**	**IgG2a**	**IgG3**
1	**-**	**+**	**+++**
2	**-**	**-**	**+++**
3	**++**	**-**	**+**
4	**-**	**-**	**+**
5	**-**	**-**	**+++**
6	**-**	**+**	**++**
7	**-**	**+**	**+++**
8	**+**	**++**	**+**
9	**+**	**+++**	**+++**
10	**+**	**+++**	**+++**
11	**+**	**+++**	**+++**
12	**++**	**+++**	**+++**

Classification according to anti-B16-F0 staining: <5% = negative, 5-20% = +; 21-50% = ++;51-100% = +++.

## Discussion

Bispecific antibodies (bsAb) are considered as a promising improvement of traditional monospecific antibodies for instance because effector functions are enhanced and the risk of drug resistance is reduced [30]. Among the many different formats, trAb are most advanced with catumaxomab being the first EMA-approved bsAb so far [21]. Here, we present preclinical data of the new trAb Surek targeting the melanoma-associated ganglioside GD2 and mCD3 on T cells. A critical point of investigation was the relatively low affinity of the antibodies’ tumor binding arm (~10^7^ M^-1^) recognizing a sugar epitope. However, in spite of this fact, Surek showed therapeutic effectiveness in the treatment of the GD2-positive B78-D14 melanoma model. The therapeutic outcome was superior to the parental monospecific antibody treatment, specific and dose-dependent with a cumulative dose of 150μg required to accomplish complete tumor rejection (Figures 3A, B, C). In comparison with the high-affinity EpCAM-specific surrogate antibody BiLu (~10^9^ M^-1^), which was evaluated in a similar melanoma model, the required therapeutic dosage is about 15–30 times higher [18,29]. This can be partially ascribed to the different affinities of the tumor binding arms. However, the finding that Surek is capable of redirecting T-cell cytotoxicity at all (EC_50_ = 70 ng/ml [0.47 nM]) may be explained by multivalent binding of redirected T cells opsonized with a multitude of Surek antibody molecules. This hypothesis implies a much higher affinity of the monovalent CD3 binding arm of Surek which was indeed confirmed by comparative FACS binding studies (Figure 1A). Depletion experiments further confirmed that the *in vivo* mode of action of Surek is also strictly dependent on T cells. Interestingly, both CD8^+^ as well as CD4^+^ T cells are essential for therapeutic effectiveness (Figure 3D) suggesting that CD4^+^ T helper cells contribute to the tumor destruction either directly or indirectly e.g. by promoting T-cell activation via cross-talk with accessory immune cells.

A considerable concern of anti-GD2 antibodies is the triggering of neurotoxicity [31]. Antibody binding to GD2 expressed as a minor constituent in normal peripheral nerves causes severe pain requiring analgetic medication [32-34]. Importantly, we did not observe any apparent signs of neurotoxicity in mice after administration of Surek. Of note, the GD2 binding arm of the therapeutic equivalent antibody Ektomab (anti-GD2 x anti-hCD3) showed no cross- reactive binding to 32 different normal human tissues including peripheral nerves. The only exception was cerebellum (unpublished data). Thus, the relatively low affinity of Ektomab obviously avoids its monospecific binding to healthy tissue and seems to be advantageous in terms of circumventing dose-limiting neurotoxicity. Moreover, the high specificity of the parental antibody Me361, which provides the GD2-binding arm of Ektomab and Surek, prevents significant cross-reactive binding with prominent ganglioside species like GM1 or GM3 [35]. We found that even with the most similar ganglioside structure GD3, there was only minimal cross-detection at high antibody concentrations of > 100 μg/ml (Figure 1B). Selecting therapeutic mAbs with an appropriate affinity is often a matter of debate that can only be answered in the clinic. For instance, high-affinity EpCAM-specific mAbs were shown to cause severe acute pancreatitis, whereas low-affinity counterparts lacked sufficient effector functions [36].

TrAb represent an attractive approach in cancer therapy because they link innate and adaptive immunity [37]. For this reason it was important to evaluate whether the trAb Surek and other trAb like BiLu [18,29] or catumaxomab [38] are equally competent in inducing long-term vaccination. Indeed, a proportion of 50% of Surek-treated long-term survivors were resistant to a lethal second tumor challenge without any further antibody injection (Figure 4A). Anti-tumor immunization was associated with the formation of tumor-reactive antibodies which, however, only marginally contribute to tumor protection as shown by plasma transfer experiments (Figure 5). In fact, the measured humoral immune response which was dominated by IgG2a and IgG3 isotypes indicates an IL-12-mediated and Th1-associated T-cell immunity [39,40]. Indeed, activation of dendritic and T cells was observed by Eissler et al. 48h after *in vivo* application of Surek accompanied by IL-12, IFN-γ and TNF release. Moreover, protective tumor-specific T cells recognizing melanoma-derived peptides like tyrosinase-related protein 2 (trp2) and gp100 were induced after immunization of mice with irradiated B78-D14 tumor cells and intact Surek antibody but not with the bsF(ab′)_2_ counterpart lacking the Fc region [41]. Thus, Surek effectively eliminates B78-D14 melanoma cells *in vivo* followed by the induction of long-term anti-tumor immunization. Of note, we did not detect autoreactive antibodies directed against the target structure GD2 indicating that Surek therapy does not break tolerance to self antigens. This is an important finding because ganglioside-directed autoimmune reactions were shown to cause severe neurological disorders in some patients [31].

Immunotherapeutic approaches are especially attractive for the treatment of immunogenic cancers like melanoma. This is underscored by the recent approval of the monoclonal antibody Ipilimumab [42], which blocks the negative T-cell regulator CTLA-4 [43]. However, severe autoimmune effects like hepatitis or enterocolitis are frequently observed in the course of Ipilimumab treatment. In contrast to nonspecific immune modulators, trAb represent a targeted immunotherapeutic approach. They offer the advantage of redirecting and activating T cells and antigen-presenting cells simultaneously at the tumor site and thus combining passive and active immunization [37]. Hence, trAb may be especially effective in the treatment of immunogenic melanoma. Our data demonstrate that the trAb Surek, which targets the melanoma-associated antigen GD2, effectively eliminates melanoma cells accompanied by the generation of an immunological memory. The typical characteristics of targeted cell destruction and induced long-term immune responses are observed for Surek. These results warrant further clinical development of its therapeutic equivalent Ektomab.

## Conclusions

The mode of action and the efficacy of trifunctional bispecific antibodies (trAb) binding with high affinity to targeted tumor-associated surface proteins like EpCAM or HER2/neu are well described. This was impressively demonstrated with the first clinical approval of the trAb catumaxomab (Removab®) in 2009. Our results obtained with Surek suggest that the concept of trAb for tumor therapy can also be extended to trAb recognizing sugar epitopes with low affinity. Surek revealed similar *in vivo* efficacy and immunizing potency like the high-affinity surrogate trAb BiLu although significant higher amounts of antibody were required. Nonetheless, there may be a therapeutic window with augmented tolerated dosages for GD2-targeting trAb due to the high tumor specificity of the expressed ganglioside. In the context of induced neurotoxicity as observed for other GD2-specific mAbs a low affinity trAb is likely to be more tolerable. In summary, our results warrant the further clinical development of GD2-targeting trAb like e.g. Ektomab.

## Competing interests

H. Lindhofer is stock holder of TRION Research and TRION Pharma GmbH. P. Ruf, B. Schäfer, J. Hess, M. Plöscher, S. Wosch, I. Suckstorff and C. Zehetmeier are employees of TRION. The other authors declare that they have no competing interest.

## Authors’ contributions

PR drafted the manuscript, designed experiments, analyzed and interpreted data and performed statistical analysis. BS contributed to the experimental design and carried out *in vivo* mouse studies. NE and SW carried out *in vivo* mouse studies and *in vitro* cytotoxicity experiments. MP conducted HPLC and mass spectrometric analysis. IS and CZ performed immunoassays and antibody quality control analysis. RM, JH and HL contributed to the design of the study, discussed the data and helped to draft the manuscript. All authors read and approved the final manuscript.
